# Histone Deacetylase Inhibitors Impair the Elimination of HIV-Infected Cells by Cytotoxic T-Lymphocytes

**DOI:** 10.1371/journal.ppat.1004287

**Published:** 2014-08-14

**Authors:** Richard Brad Jones, Rachel O'Connor, Stefanie Mueller, Maria Foley, Gregory L. Szeto, Dan Karel, Mathias Lichterfeld, Colin Kovacs, Mario A. Ostrowski, Alicja Trocha, Darrell J. Irvine, Bruce D. Walker

**Affiliations:** 1 The Ragon Institute of Massachusetts General Hospital, Massachusetts Institute of Technology, and Harvard University, Boston, Massachusetts, United States of America; 2 Koch Institute for Integrative Cancer Research, MIT, Cambridge, Massachusetts, United States of America; 3 Department of Biological Engineering, MIT, Cambridge, Massachusetts, United States of America; 4 Massachusetts General Hospital, Boston, Massachusetts, United States of America; 5 The Maple Leaf Medical Clinic, Toronto, Ontario, Canada; 6 Department of Medicine, University of Toronto, Toronto, Ontario, Canada; 7 Li Ka Shing Medical Institute, St. Michael's Hospital, Toronto, Ontario, Canada; 8 Howard Hughes Medical Institute, Chevy Chase, Maryland, United States of America; Emory University, United States of America

## Abstract

Resting memory CD4^+^ T-cells harboring latent HIV proviruses represent a critical barrier to viral eradication. Histone deacetylase inhibitors (HDACis), such as suberanilohydroxamic acid (SAHA), romidepsin, and panobinostat have been shown to induce HIV expression in these resting cells. Recently, it has been demonstrated that the low levels of viral gene expression induced by a candidate HDACi may be insufficient to cause the death of infected cells by viral cytopathic effects, necessitating their elimination by immune effectors, such as cytotoxic T-lymphocytes (CTL). Here, we study the impact of three HDACis in clinical development on T-cell effector functions. We report two modes of HDACi-induced functional impairment: i) the rapid suppression of cytokine production from viable T-cells induced by all three HDACis ii) the selective death of activated T-cells occurring at later time-points following transient exposures to romidepsin or, to a lesser extent, panobinostat. As a net result of these factors, HDACis impaired CTL-mediated IFN-γ production, as well as the elimination of HIV-infected or peptide-pulsed target cells, both in liquid culture and in collagen matrices. Romidepsin exerted greater inhibition of antiviral function than SAHA or panobinostat over the dose ranges tested. These data suggest that treatment with HDACis to mobilize the latent reservoir could have unintended negative impacts on the effector functions of CTL. This could influence the effectiveness of HDACi-based eradication strategies, by impairing elimination of infected cells, and is a critical consideration for trials where therapeutic interruptions are being contemplated, given the importance of CTL in containing rebound viremia.

## Author Summary

The advent of antiretroviral therapy has greatly improved the prognosis for HIV-infected individuals with access to care. However, current therapies are unable to cure infection, committing treated individuals to a lifetime of medication with significant economic burden. Furthermore, it has become clear that antiretroviral therapy does not completely restore health, leaving treated HIV-infected individuals at increased risk of cardiovascular disease, neurological disorders, and other health issues. Thus, there is a need to develop therapies capable of curing HIV infection. It is thought that, to be successful, curative strategies will need to combine a means to flush the virus out of the latently-infected cells in which it hides, with a means to kill these unmasked targets. A front-running approach proposes to use a class of drugs called histone deacetylase inhibitors (HDACis) as flushing agents, with cytotoxic T-lymphocytes (CTL, or killer T-cells) to purge viral reservoirs. Here, we uncover an unexpected negative interaction between these two agents, whereby HDACis suppress the ability of CTL to kill HIV-infected cells. This interaction has the potential to limit the effectiveness of combining CTL with HDACis in flush and kill approaches to HIV eradication, and should be considered in the prioritization and optimization of potential curative strategies.

## Introduction

Antiretroviral therapy (ART) is capable of durably suppressing viremia in HIV-infected subjects, but is unable to cure infection. The financial and psychological burden of lifelong therapy, as well as a growing appreciation for co-morbidities that occur in HIV-infected individuals on long-term therapy, such as cardiovascular disease and neurocognitive disorders, have led to the prioritization of HIV cure research [1], [Deeks2]. The best understood, and perhaps most obstinate, barrier to eradicating infection is the existence of a pool of infected resting memory CD4^+^ T-cells [3]–[5]. By virtue of their quiescent state, these cells are not thought to express HIV antigens, rendering them invisible to the immune system. These cells are very long-lived, with an estimated half-life of 44 months, suggesting that 60 years of uninterrupted ART would be required for full decay of the reservoir [6]. As the reservoir almost certainly replenishes itself through ongoing rounds of re-infection and homeostatic proliferation, it is unlikely that current ART regimens could cure an individual within a lifetime [7], [8].

Such theoretical and experimental analyses have led to the consensus that the eradication of HIV from an infected individual will require a means for actively depleting the resting CD4^+^ T-cell reservoir, most likely to be achieved by inducing viral expression that could trigger immune-mediated clearance of infected cells. While a variety of compounds have been shown to reactivate virus from CD4^+^ T-cells, a class of drugs known as histone deacetylase inhibitors (HDACis) has emerged as the front-runner and a number of these, including vorinostat (suberoylanilide hydroxamic acid or SAHA), romidepsin, and panobinostat, have entered into HIV clinical trials aimed at testing their abilities to reduce or eradicate viral reservoirs in the context of ART [9]–[11] (reviewed in [12]).

It was initially thought that the reactivation of latent HIV by HDACis would be sufficient to eliminate infected cells through viral cytopathic effects. Recent data, showing that *in vitro* treatment of patient PBMCs with 500 nM SAHA failed to lead to a reduction in inducible viral reservoirs, suggests that this may not be the case, and that immune effectors, such as HIV-specific cytotoxic T-lymphocytes (CTL), natural killer (NK) cells, or immunotoxins will likely be needed to recognize and eliminate these exposed target cells in so called ‘flush-and-kill’ strategies [13]. Notably, in this same study, CD8^+^ T-cells freshly isolated from ART-treated HIV-infected patients could eliminate infected cells in a primary cell model of latency only if pre-stimulated with peptides and IL-2 *ex vivo* [13], highlighting that, in the case of CTL-based flush-and-kill strategies, the functional state of virus-specific CD8^+^ T-cells will be important for reservoir elimination.

In evaluating strategies predicated upon coordinating CTL with latency-reversing drugs it is critical to consider potential side effects of these drugs on CTL function. This is particularly true in the case of HDACis, which are known to exert potent and diverse effects on both the innate and adaptive immune system (reviewed in [14] and [15]). A number of HDACis, including SAHA, have been shown to suppress the production of inflammatory cytokines by both T-cells and innate immune cells, *in vitro* and *in vivo* [16]–[21]. SAHA, romidepsin, and other HDACis have also been shown to interfere with the differentiation of monocytes into dendritic cells (DCs), as well as to block the ability of DCs to upregulate CD1a, CD80, CD83 and other co-stimulatory molecules, resulting in impaired priming of T-cells [22]–[26]. These immunosuppressive activities of HDACis have been associated with therapeutic benefits in murine models of graft-versus-host disease (GVHD) and autoimmune/inflammatory disorders such as autoimmune lymphoproliferative syndrome, experimental autoimmune encephalomyelitis (EAE, a model of multiple sclerosis), and diabetes mellitus [27]–[32].

While it is tempting, based on this body of evidence, to generally characterize HDACis as immunosuppressive agents, this would not be an accurate assessment. The HDACi panobinostat (LBH589), which is also in clinical trials for HIV eradication (CLEAR trial, ClinicalTrials.gov Identifier NCT00256139), has been reported to enhance T-cell activation *in vivo*, resulting in elevated serum Th1 cytokines and accelerated progression in a murine model of graft-versus-host disease [33]. Even within an individual, the effects of a given HDACi have been reported to be divergent depending upon the particular facet of the immune response being studied. For example, in a murine model of allogeneic bone marrow transplantation it has been shown that SAHA decreased levels of serum cytokines and GVHD while having no effect on donor T-cell proliferation or killing of host cells [20]. In a second example, co-administration of the HDACi MS-275 with a viral-vectored vaccine served to suppress the immune response to the vector, while enhancing the response to the viral vector insert and suppressing autoimmune pathology [34].

This diversity of effects of HDACis on immune cells is likely rooted in two sources. First, there are 18 different HDAC enzymes in humans, divided into four different families. Different HDACi drugs interact with different subsets of these enzymes, depending upon the dose being used [35]. Second, in addition to altering histone acetylation status, and thus chromatin structure, HDACis can interact with multiple transcription factors, including NF-κB, AP-1, and others either by interfering with co-repressor HDAC enzymes that are recruited to transcription factor binding sites resulting in enhanced transcription, or by directly blocking deacetylation of the transcription factor itself [36]–[40]. Foxp3, for example, requires acetylation of lysine residues for maximal activation. HDACis, by preventing deacetylation of Foxp3, enhance its activity and thus boost the numbers and function of Tregs *in vivo* [41]. Other nonhistone proteins that serve as direct HDAC substrates include p53, GATA-1, STAT3, and STAT5 [42], [43]. This high degree of complexity, both in terms of immunological outcomes and underlying mechanisms, necessitates that HDACis be studied in a context that is matched to their intended utility. Thus, there is a need to understand the impact of HDACis being taken forwards in flush-and-kill eradication strategies on the abilities of HIV-specific CTL to eliminate infected target cells.

Here, we report a series of experiments designed to assess the effects of the HDACis currently being tested in HIV eradication clinical trials on the *in vitro* function of virus-specific T-cells. We show that, while the HDACis tested did not exhibit detectable toxicity to *ex vivo* bulk CD8^+^ T-cells over the doses and time-courses tested, romidepsin and, to a lesser extent, panobinostat exhibited delay toxicity to activated CD8^+^ T-cells and to CTL clones. Each of the HDACis tested rapidly suppressed the production of IFN-γ from PMA/ionomycin by *ex vivo* stimulated CD8^+^ and CD4^+^ T-cells at an early time-point not associated with losses in viability. The production of IFN-γ in response to peptides representing viral epitopes was rapidly and durably suppressed by treatment of both CTL clones and *ex vivo* CD8^+^ T-cells with HDACis. Treatment with romidepsin abrogated the proliferation of HIV-Gag- and CMV-pp65-specific CD8^+^ and CD4^+^ T-cells, while panobinostat and SAHA only significantly impaired proliferation of CMV-pp65-specific CD8^+^ T-cell responses. Of principal importance, we observed that each of the HDACis tested exhibited significant impairment of the abilities of CTL clones to eliminate HIV-infected target cells. These results indicate that HDACis can exert negative effects on the CTL that may be needed for flush-and-kill approaches to eradication.

## Results

### Effects of HDAC Inhibitors on T-Cell Viability over a 21 Hour Time-Course

**Figure 1:**
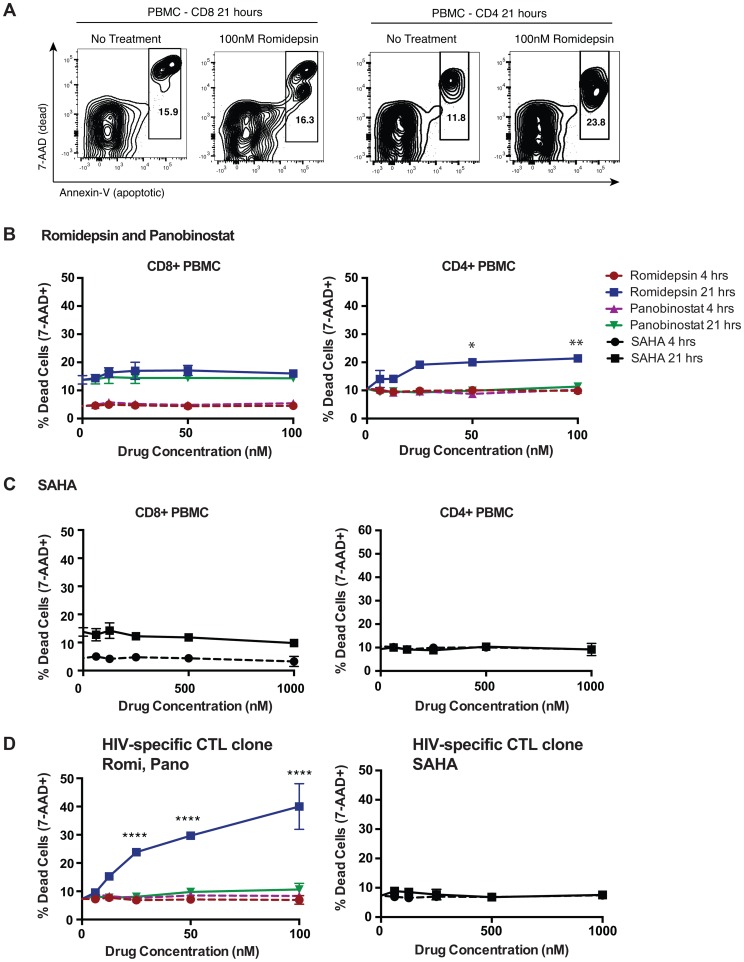
Effects of HDACis on the viability of PBMC CD8^+^ and CD4^+^ T-cells. **A–C**. PBMC were treated with HDACi drugs at the indicated concentrations for either 4 or 21 hours (drugs left in) then stained with Annexin-V-Fitc (stains phosphatidyl serine on apoptotic cells), 7-AAD (stains DNA of dead cells), CD4 pacific blue, and CD8 alexa-fluor 700 and analyzed by flow cytometry. **A**. Shown are representative flow cytometry data indicating gating on dead (Annexin-V^+^7-AAD^+^) and dying (Annexin-v^bright^7-AAD^dim/−^) cells. **B, C**. Shown are summary data for treatments PBMC gated on CD8^+^ cells (left panel) or CD4^+^ cells (right panel) with romidepsin and panobinostat (**B**) or with SAHA (**C**) at the indicated doses for the indicated times. P values for comparisons between the multiple doses of romidepsin tested and the untreated condition were calculated by Kruskal-Wallis test and found to be significant for *ex vivo* CD4^+^ T-cells (p = 0.0080). Post-hoc Dunn's multiple comparison tests were performed for each of these and the indicated p values are adjusted for multiple testing. Other drug treatment conditions and cell types were not significant by Kruskal-Wallis tests. **D**. The effects of HDACi treatment on the viability of an HIV-Gag-SLYNTVATL-specific T-cell clone were determined in the same manner as for PBMC, including use of the same statistical tests. Error bars represent SD. * p<0.05, ** p<0.01, *** p<0.001, **** p<0.0001.

As a prerequisite to assessing the effects of HDACis on T-cell function, we first measured the impact of HDACi treatment on T-cell viability. Freshly isolated PBMC from an HIV-uninfected donor were treated separately with serial dilutions of romidepsin, panobinostat or SAHA at pharmacologically relevant concentrations (see Methods for justification of concentration ranges selected). At 4 and 21 hour time-points cells were stained with 7-Aminoactinomycin D (7-AAD, stains DNA of dead cells) and fluorochrome-conjugated annexin-V (stains phosphotidyl serine, an early apoptosis marker) and analyzed by flow cytometry. At 4 hours of treatment we did not observe significant loss of cell viability under any of the treatment conditions tested (**Fig. 1A–C**). At 21 hours of treatment we continued to observe a lack of impact of HDACi treatment on CD8^+^ T-cell viability, but did observe a significant induction of CD4^+^ T-cell death at doses of 50 and 100 nM, reaching 21.4±0.9% dead cells with 100 nM romidepsin, (p = 0.0038 compared to no treatment)) (**Fig. 1A–C**). Neither SAHA nor panobinostat was associated with loss of T-cell viability at 21 hours.

The effects of HDACis on cell viability were also assessed using pulse-wash experimental setup with cryopreserved PBMC from two HIV-infected ARV-treated subjects. Cells were treated with drugs for 6 hours, washed, plated in fresh medium, and then viability was assessed at 16, 21, and 28 hours by 7-AAD staining and flow cytometry. Beginning at 21 hours, significant losses in viability were only observed in CD4^+^ T-cells upon treatment with higher concentrations of romidepsin or panobinostat, and these increased by 28 hours. A significant loss in CD8^+^ T-cell viability was only observed with 100 nM romidepsin in cells from one of the two subjects at the 28 hour time-point (**Supporting Fig. S1**). No significant losses in viability were observed in association with treatment with up to 1,000 nM of SAHA in this experiment (data not shown). Thus, while relatively high doses of romidepsin and panobinostat negatively impacted the viability of CD4^+^ T-cells, no effects on bulk CD8^+^ T-cell viability were observed out to 21 hours.

### Romidepsin and Panobinostat Are Disproportionately Toxic to Activated T-Cells, Including CTL Clones

**Figure 2:**
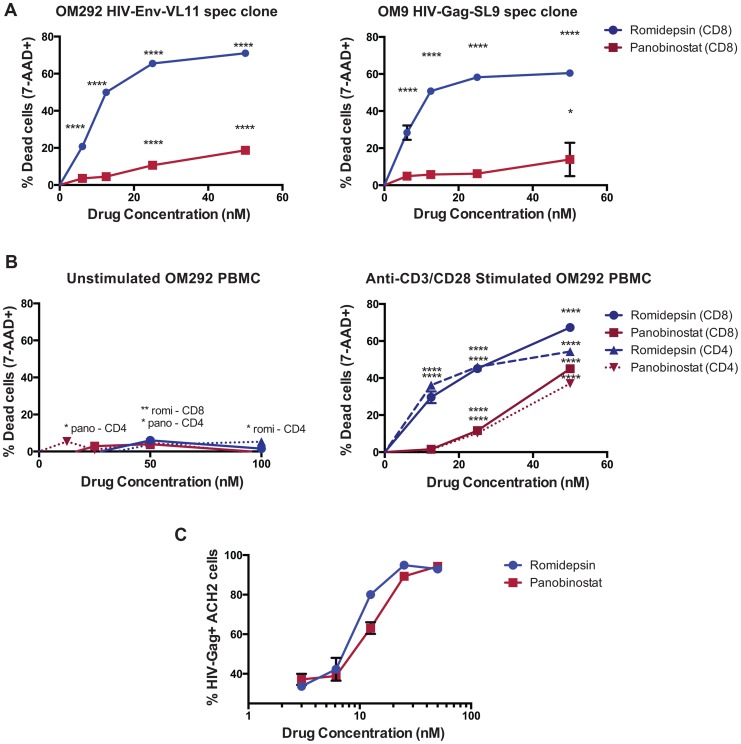
Romidepsin and panobinostat are disproportionately toxic to activated CD4^+^ and CD8^+^ T-cells. **A**. HIV-Env-VL11 and HIV-Gag SL9 specific CD8^+^ CTL clones were isolated from subjects OM292 and OM9 respectively, and exposed to HDACis at the indicated concentrations for 4 hours. Cells were then washed to remove drugs and replated in fresh R10 medium supplemented with 50 U/ml IL-2 for an additional 17 hours. Cell viability was measured by 7-AAD staining and flow cytometry. **B**. PBMC from subject OM292 either directly *ex vivo* (left panel) or following 48 hours of stimulation with anti-CD3 and anti-CD28 (right panel) were exposed to HDACis at the indicated concentrations for 4 hours. Cells were then washed to remove drugs and replated in fresh R10 medium. Cell viability was then measured by 7-AAD staining and flow cytometry. For **A** and **B**, shown are graphs of mean values taken from triplicate wells ± SEM following subtraction of background (% 7-AAD+ in no drug controls). P values were calculated by two-way ANOVA with Dunnett's multiple comparison test (comparing to the no drug control) * p<0.05, ** p<0.01, *** p<0.001, **** p<0.0001. C. In parallel to the viability assays shown in **A** and **B**, ACH2 cells were treated with HDACis for 4 hours, then washed and replated in fresh medium. Shown are mean ± SEM values for % HIV-Gag^+^ cells as measured in triplicate by intracellular staining and flow cytometry.

We also tested the effects of HDACis on CTL clones. Following 4 hours of exposure we did not observe losses in cell viability at the doses tested (**Fig. 1D**). However, 21 hours of sustained exposure led to significant losses in CTL viability with as little as 25 nM romidepsin, but not with panobinostat or SAHA (**Fig. 1D**). To further explore this, we tested the effects of a 4 hour pulse/wash exposure to romidepsin, panobinostat, and SAHA on the viability of the same clone, and on an additional clone, specific for HIV-Env-VPVWKEATTTL. We observed high levels of CTL death with romidepsin treatment that were significant with as little as 6 nM of drug (**Fig. 2A**). Substantially less cell death was observed with equivalent concentrations of panobinostat, however significant effects were still detected at 25 and 50 nM of drug for the Env-specific CTL clone, and at 50 nM for the Gag-specific CTL clone. No significant losses in viability were observed with up to 1,000 nM of SAHA (data not shown). We posited that the differential level of toxicity of romidepsin and panobinostat towards CTL, versus *ex vivo* T-cells, may be attributable to the greater activation state of the former. To test this, PBMC from an ARV-treated HIV-infected subject were thawed and stimulated with anti-CD3 of anti-CD28 antibodies for 48 hours. These cells were pulsed with the indicated doses of HDACis for 4 hours, drugs were washed out, cells were cultured for an additional 17 hours, and viability was assessed. Parallel treatments were performed on PBMC directly after thawing without stimulation. As in previous experiments, we observed only low levels of cell death in HDACi-treated unstimulated T-cells (**Fig. 2B**, left panel). We observed strikingly higher levels of cell death in stimulated CD8^+^ and CD4^+^ T-cells (**Fig. 2B**, right panel). As with CTL clones, romidepsin exhibited substantially greater toxicity to activated cells than panobinostat, particularly at the lower concentrations. In parallel to the viability experiments, to confirm the activity of our drugs, we tested these same stocks of romidepsin and panobinostat for their ability to reverse HIV latency in the ACH2 cell line model [44], and observed very similar dose response curves (**Fig. 2C**). Thus, romidepsin and panobinostat are disproportionately toxic to activated T-cells, including those that have received short-term (48 hour) TCR stimulation. Romidepsin was substantially more toxic to CTL and activated T-cells than panobinostat, despite similar potencies of latency-reversal by these two drugs as measured against a cell line model.

### HDAC Inhibitors Impair IFN-γ Production from T-Cells before Losses in Viability Occur

**Figure 3:**
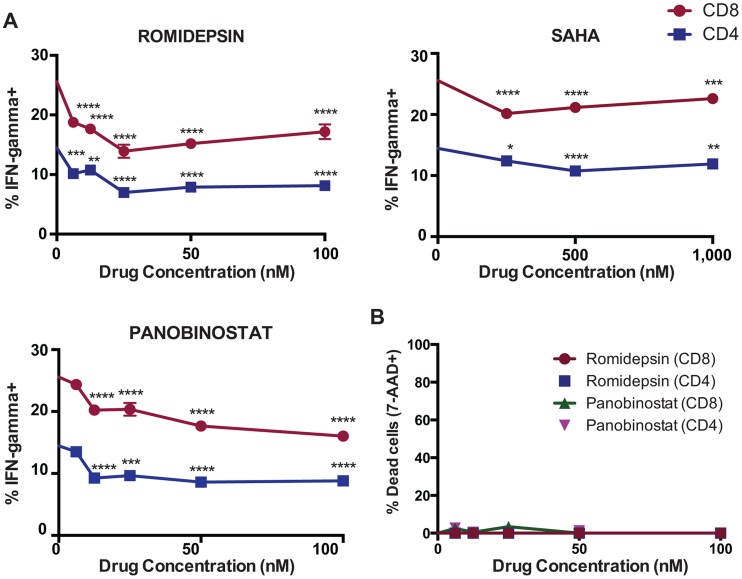
HDAC inhibitors impair IFN-γ production from PMA/ionomycin stimulated CD4^+^ and CD8^+^ T-cells. **A, B**. PBMC from subject OM292 were exposed to HDACis for 4 hours. Cells were then washed and cultured in fresh medium with PMA/ionomycin for an additional 5 hours in the presence of brefeldin A. **A**. IFN-γ production in CD4^+^ and CD8^+^ T-cells was measured by intracellular cytokine staining flow cytometry. Shown are mean ± SEM values. P values were calculated by two-way ANOVA with Dunnett's multiple comparison test (comparing to the no drug control) * p<0.05, ** p<0.01, *** p<0.001, **** p<0.0001. **B**. Viability in CD4^+^ and CD8^+^ T-cells was measured by Annexin-V and 7-AAD staining flow cytometry. Shown are mean ± SEM values.

Next, we measured the effects of exposure to HDACis on IFN-γ production from *ex vivo* T-cells. PBMC from an ARV-treated HIV-infected subject were exposed to the indicated concentrations of HDACis for 4 hours, and then stimulated with PMA/ionomycin for 5 hours. We observed that romidepsin, panobinostat, and SAHA each significantly suppressed the production of IFN-γ by both CD8^+^ and CD4^+^ T-cells at these pharmacologically relevant concentrations (**Fig. 3A**), without any significant losses in cell viability (**Fig. 3B**). Thus, while in previous experiments treatment with romidepsin and, to a lesser extent panobinostat, resulted in the death of activated T-cells at relatively late time-points post-exposure, treatment with all three HDACis was also found to suppress IFN-γ production at early time-points, before any losses in viability were detectable.

### HDAC Inhibitors Exert Rapid and Durable Suppression of IFN-γ Production from HIV-Specific Antigen-Stimulated CD8^+^ T-Cells

**Figure 4:**
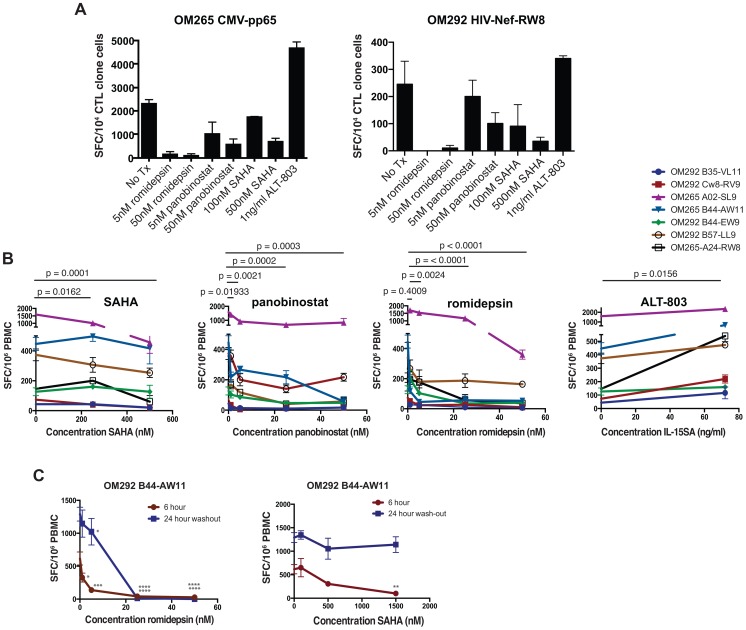
HDAC inhibitors impair IFN-γ production from antigen-stimulated CD8^+^ T-cells. **A, B**. PBMC or CTL clones from chronically HIV-infected ARV-treated subjects (OM265 and OM292) were cultured for 2 hours with the indicated drugs and then plated for IFN-γ ELISPOT assays with 12 hour peptide stimulation periods. **A**. Treated CTL clones were stimulated with overlapping 15mer peptides spanning the CMV-pp65 protein (left panel) or the MHC-I-A24 restricted HIV-Nef peptide ‘RW8’ (right panel). Each treatment condition was tested in duplicate. Shown are mean SFC/10^4^ CTL clone cells for each condition with error bars representing SEM. **B**. Experiments analogous to those depicted in **A** were performed for 8 different optimal HIV CD8^+^ T-cell epitopes (5 for OM292 and 1 for OM265). Shown are summary data for all 8 responses depicting the mean SFC/10^6^ PBMC (of triplicate wells) under different treatment conditions. For SAHA, panobinostat, and romidepsin conditions statistical significance for each drug was evaluated using two-way ANOVA tests, and p values were calculated using Dunnett's multiple comparison test to account for the use of three different drug concentrations. For IL-15SA p values were calculated using the Wilcoxon matched pair test. **C**. PBMC from subjects OM265 and OM292 were treated with SAHA or romidepsin at the indicated concentrations for 2 hours and then either: stimulated with peptides for 6 a hour ELISPOT assay, or washed, cultured for 24 hours in the absence of drugs, and stimulated with peptides for a 12 hours ELISPOT assay. P values were calculated by two-way ANOVA with Dunnett's multiple comparison test (comparing to the no drug control) * p<0.05, ** p<0.01, *** p<0.001, **** p<0.0001.

We next measured the effect of exposure to HDACis on cytokine production from virus-specific CD8^+^ T-cells using IFN-γ ELISPOT assays. CMV-pp65- and HIV-Nef-RW8-specific CD8^+^ T-cell clones isolated from two ARV-treated HIV-infected subjects were treated with the indicated pharmacologically relevant concentrations of romidepsin, panobinostat, or SAHA. As a point of contrast we also included conditions where cells were pre-treated with ALT-803, an IL-15 superagonist expected to increase IFN-γ ELISPOT responses (see Methods). Following 2 hours of treatment with the designated concentrations of HDACis or ALT-803, CTL clones were cultured with autologous BLCL targets in ELISPOT plates, and stimulated for 12 hours with cognate peptides (without washing to remove HDACis or ALT-803). For both CTL clones, we observed that treatment with romidepsin, panobinostat, or SAHA resulted in reduced frequencies of T-cell responses, measured in spot-forming cells (SFCs) as compared to untreated controls (**Fig. 4A**). Inhibition of cytokine production was dose dependent, and particularly marked for romidepsin, where IFN-γ production was essentially abolished. In contrast, the IL-15 superagonist ALT-803 enhanced frequencies of IFN-γ producing cells.

We then tested the effects of these drugs on IFN-γ production from CD8^+^ T-cells directly *ex vivo* from ARV-treated HIV-infected subjects. PBMC were treated with HDACis or ALT-803 for 2 hours then peptides representing optimal CD8^+^ T cell epitopes that been previously mapped in these subjects were added (without washing to remove HDACis). We observed that, as with CTL clones, all three of the HDACis tested suppressed HIV-specific IFN-γ production from *ex vivo* samples in a dose-dependent manner (**Fig. 4B**). In contrast, treatment with IL-15 superagonist resulted in a significant increase in IFN-γ production.

The above ELISPOT assays were based on 16 hour peptide stimulation periods, with the final readout representing the cumulative IFN-γ production over this period. Based on the data presented in **Figs. 2** **and** **3** we postulated that two factors may have contributed to this net loss in IFN-γ production: i) the rapid suppression of IFN-γ production from viable cells at early time-points post-stimulation and ii) particularly in the case of romidepsin, the death of HDACi exposed stimulated cells at later time-points. The involvement of both of these factors would result in the HDACi suppressive effect being both rapid in onset and durable, as HDACi wash-out periods would not reverse losses in IFN-γ production caused by the death of antigen-specific cells. We tested this by probing the kinetics of this suppressive effect. To test the contributions of the former, we performed short-term treatments, with 2 hour exposures of *ex vivo* PBMC to either romidepsin or SAHA, followed by 6 hour stimulations with the HIV-Gag optimal epitope AW11. To test the latter, we exposed PBMCs with romidepsin or SAHA for 2 hours, washing to remove the drug, culturing for 24 hours in the absence of drug, and then stimulating with peptide for 12 hours in ELISPOT assays (24 hour washout). We observed the dose-dependent impairment of IFN-γ production in both the 6 hour stimulation and 24 hour wash-out assays following treatment with romidepsin (**Fig. 2C**). With SAHA treatment, rapid impairment was observed in 6 hour ELISPOT assays, but this was largely normalized following a 24 hour washout period. Panobinostat was not tested in this assay. Thus each of the HDACis studied suppress IFN-γ production from HIV-specific CD8^+^ T-cells (both *ex vivo* and CTL clones). This effect was rapid in onset for both romidepsin and SAHA, and in the case of romidepsin was sustained following removal of drug (for at least 24 hours). While we were unable to directly measure the viability of the antigen-specific T-cells at the end of these ELISPOT assays, our data with anti-CD3 stimulated cells (**Fig. 2B**) support the idea that the inability of these T-cells to recover over a 24 hour period may reflect the selective depletion of these cells by the combination of antigenic stimulation and romidepsin treatment.

### HDAC Inhibitors Differentially Impair Proliferation of Virus-Specific CD8^+^ T-Cells

**Figure 5:**
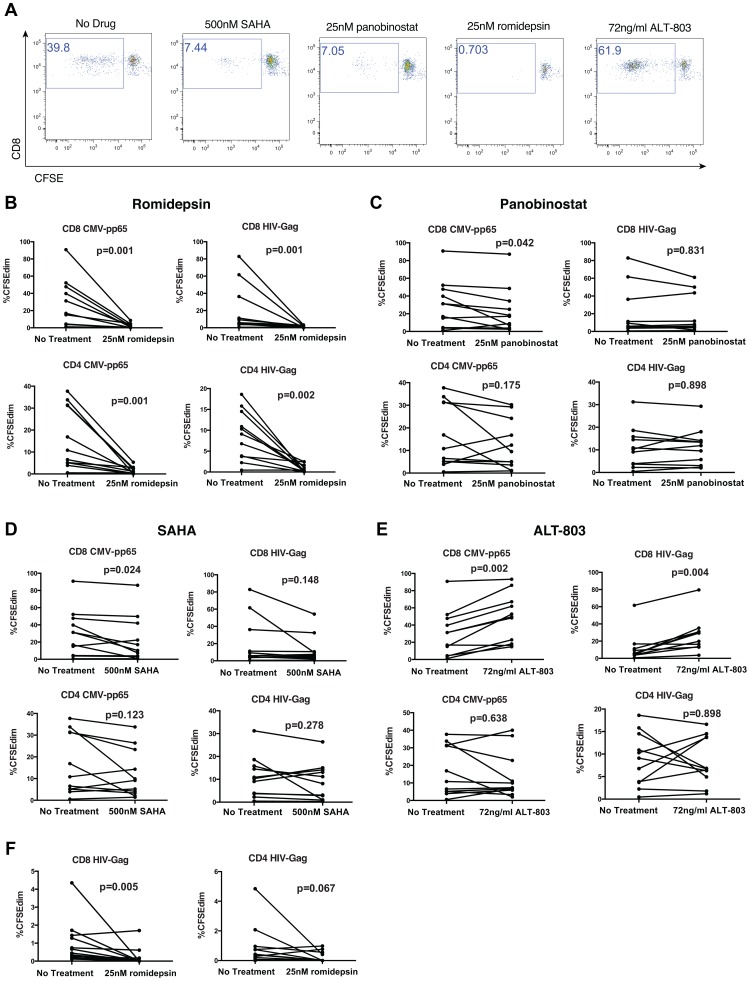
HDAC inhibitors impair proliferation of virus-specific T-cells. PBMC from 11 chronically HIV-infected subjects on suppressive ARV therapy were labeled with CFSE and treated with the indicated drugs for 4 hours. HIV-Gag or CMV-pp65 peptide pools were then added to final concentrations of 1 µg/ml/peptide. 48 hours later, cells were washed thoroughly, re-suspended in medium with 20 U/ml IL-2, and cultured for an additional 5 days. Cells were then stained with a viability dye and antibodies to CD3, CD8, and analyzed by flow cytometry. **A**. Shown are representative flow cytometry data of a CMV-pp65 response gated on viable, CD3^+^CD8^+^ lymphocytes depicting CFSE (diminution indicates proliferation) – x-axis by CD8 – y-axis. **B–E**. Summary data depicting frequencies of CFSEdim cells within viable, CD3^+^CD8^+^ or CD3^+^CD8^−^ (CD4^−^ T-cell) lymphocyte populations. **F**. Shown are data analogous to **B–E**, but from a separate experiment where peptides were added back with fresh medium following HDACi wash-out at 48 hours. P values were calculated by the Wilcoxon matched-pairs signed rank test.

Next we tested the effects of HDACis on the proliferation of CD8^+^ T-cells in response to HIV-Gag and CMV-pp65 peptide pools using a standard CFSE proliferation assay. We reasoned that *in vivo* there would be a delay between the time of HDACi administration and the expression of HIV antigens, during which antigen-specific T-cells would be exposed to drugs. For a single round of HDACi administration, cells that recognized induced HIV antigens would have a chance to recover from any effects of HDACis upon drug clearance, and potentially proliferate. To model this scenario, PBMCs were exposed to HDACis or ALT-803 for 4 hours prior to addition of specific peptides. Cells were then co-cultured with HDACis and peptide for 48 hours, washed extensively, and resuspended in fresh medium with 20 U/ml IL-2 for an additional 5 days. We observed the proliferation of CD8^+^ T-cells in response to CMV-pp65 peptide pools (**Fig. 5A**), and a lesser degree of proliferation in response to HIV-Gag peptide pools (data not shown). Romidepsin, at the pharmacologically relevant dose of 25 nM, abrogated proliferation of CD8^+^ T-cells in response to both peptide pools (**Fig. 5A, B**). CD8^+^ T-cells treated with 25 nM panobinostat or 500 nM SAHA (also pharmacologically relevant concentrations, see Methods) exhibited significantly reduced proliferation in response to CMV-pp65 than untreated controls (panobinostat – p = 0.042, SAHA – p = 0.024, **Fig. 5C and D**). Statistically significant effects of panobinostat or SAHA on the proliferation of CD8^+^ T-cells in response to HIV-Gag were not observed. However, our sensitivity to detect such an effect may have been limited by the relatively low levels of baseline proliferation in response to Gag. As a point of contrast, the IL-15SA ALT-803 significantly enhanced the proliferation of CD8^+^ T-cells in response to both CMV-pp65 and HIV-Gag peptide pools.

In these experiments we also examined the effects of HDACis on CD4^+^ T-cell proliferation (**Fig. 5B, C, D**). As with CD8^+^ T-cells, we observed that romidepsin abrogated proliferation of CD4^+^ T-cells in response to either HIV-Gag or CMV-pp65 peptide pools. A consistent effect on CD4^+^ T-cell proliferation was not observed for either panobinostat or SAHA in response to either HIV-Gag or CMV-pp65.

To further test the durability of the inhibition of T-cell proliferation by romidepsin we performed a variation on the above experiment where, after washing out drugs and peptides at 48 hours, additional peptides were added back along with the fresh medium. We observed significant inhibition of proliferation of CD8^+^ T-cells (p = 0.005), and a trend towards reduced proliferation of CD4^+^ T-cells (p = 0.067) in response to HIV-Gag peptides, despite 5 days of antigenic stimulation in the absence of drug (**Fig. 5F**). As with the IFN-γ ELISPOT results, the selective depletion of antigen-specific cells by stimulation in the presence of romidepsin may explain the lack of recovery following a romidepsin wash-out period. Thus, HDACis differentially affect the proliferation of T-cells in response to viral peptides, with romidepsin abrogating proliferation of CD8^+^ and CD4^+^ T-cells in response to either CMV-pp65 or HIV-Gag peptides, while panobinostat and SAHA only exhibited significant suppression of CMV-pp65-specific CD8^+^ T-cells.

### HDAC Inhibitors Impair CTL Killing of HIV-Infected CD4^+^ Target Cells

**Figure 6:**
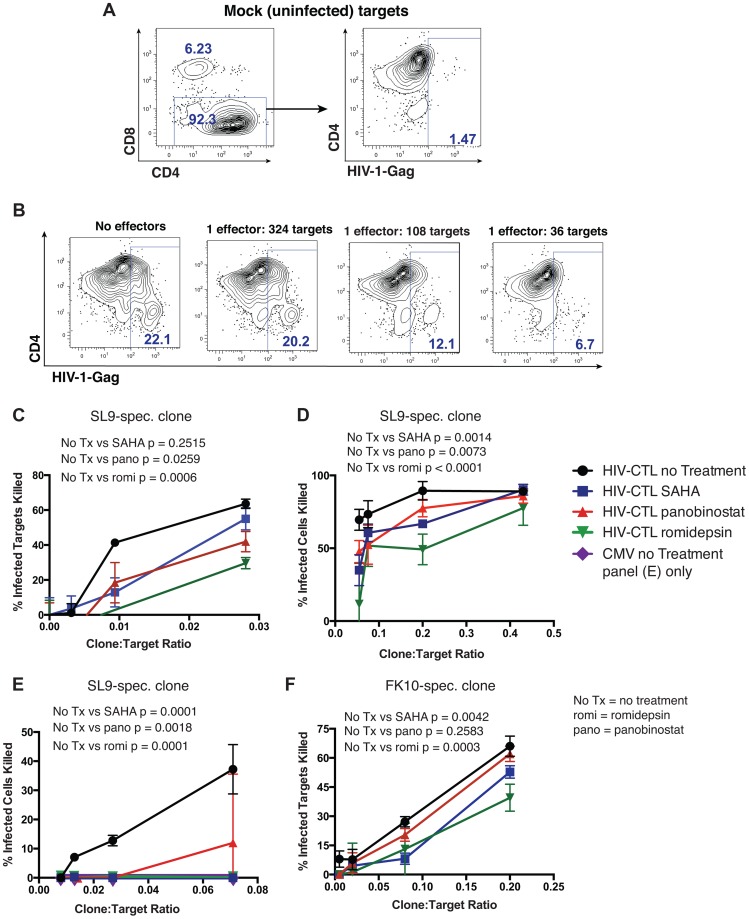
HDAC inhibitors impair CTL killing of HIV-infected primary CD4^+^ cells. CD4^+^ T-cells were enriched from HIV-uninfected A02^+^ donors and either infected with HIV JR-CSF or maintained as mock infected controls. 24 hours post-infection, these target cells were co-cultured with an HIV-Gag- or CMV-pp65-specific CTL clones at the indicated effector (clone)∶target (CD4^+^ cell) ratios for 16 hours. Cells were then stained with fluorochrome conjugated antibodies to CD8, CD4, and HIV-Gag (intracellular staining) and analyzed by flow cytometry. **A**. Shown is the gating strategy utilized for killing assays, with the placement of Gag^+^ and Gag^+^CD4^dim^ gates determined based on a mock-infected control (right panel). **B**. Data from a representative experiment with indicated effector∶target ratios of an HIV-Gag-SLYNTVATL-specific T-cell clone. Panels **C-F** utilize total Gag^+^ cells and calculate a % Infected Cells Killed as (%Gag^+^ No Effectors - %Gag^+^ at Given E∶T ratio)/%Gag^+^ No Effectors*100, ex. in panel **B** %Infected Cells Killed at 1∶36 ratio = (22.1−6.7)/22.1*100 = 69.7%. **C–F**. Shown are the results from 4 independent experiments. In **C** CTL were treated with romidepsin or panobinostat at 50 nM or with SAHA at 500 nM for 2 hours, and then co-cultured with target cells without a washing step. In **D–F** CTL were treated with romidepsin or panobinostat at 25 nM or with SAHA at 500 nM for 6 hours, then washed thoroughly before initiating a 16 hour co-culture with target cells. **D** and **E** both utilize an HIV-Gag-SLYNTVATL-specific CTL clone at high and low E∶T ratios respectively. **E** utilizes an HIV-Gag-FLGKIWPSHK-specific CTL clone. **C–F**. Shown are means ± SEM of data from triplicate wells. P values were calculated by two-way ANOVA with Tukey's multiple comparison test.

Of greatest relevance to flush and kill approaches to HIV eradication is whether HDACis impact the ability of CTL to kill HIV-infected target cells. We tested this using several variations of an infected-cell elimination assay. HLA-A02^+^ primary CD4^+^ T-cells were infected with HIV JR-CSF and then co-cultured with HLA-A02-restricted HIV-specific CTL clone of defined epitope specificity that had been pre-treated for 2 hours with HDACi drugs at various effector: target ratios. Following a 16 hour co-culture in the continued presence of HDACis, we determined the frequency of HIV-infected cells by flow cytometry and compared this to co-cultures of untreated CTLs and infected CD4^+^ cells in the absence of HDACi. Representative control data using an untreated HIV-Gag-SLYNTVATL-specific CTL clone are presented in **Fig. 6A,B**. As shown in **Fig. 6C**, co-cultures treated with pharmacologically relevant doses of panobinostat and romidepsin showed significantly reduced elimination of infected cells relative to untreated cultures. Treatment with SAHA was associated with reduced elimination of infected cells at clone∶target ratios of 1∶100 and 1∶35 (mean ± SD of proportion killed, no treatment - 41.4±1.9 vs SAHA - 12.9±14.4 at 1∶100, and no treatment – 63.6±4.7 vs SAHA 55.0±11.0), but this was not statistically significant after correction for multiple testing in this experiment (p = 0.25).

In order to isolate the effects of HDACis on CTL from potential effects on target cells, we performed variations of the above experiment where we pre-treated CTL with drugs at the above concentrations for 4 hours and then washed to remove the drugs prior to co-culturing with targets. Using an HIV-Gag-SLYNTVATL-specific CTL clone we tested two different ranges of clone∶target ratios (**Figs. 6D** (high range), **6E** (low range)). In both of these experiments we observed significantly impaired elimination of HIV-infected target cells by CTL that had been pre-treated with SAHA, panobinostat, or romidepsin, with the most pronounced effect at low effector to target ratios. To control for non antigen-specific elimination of infected cells, we co-cultured infected target cells with a CMV-pp65-specific CTL clone in parallel to the HIV-Gag-specific CTL clone over the range of effector∶target ratios tested. We observed a lack of elimination of infected target cells with this CMV-pp65-specific clone (data-points are along the x-axis, **Fig. 4E**).

To further corroborate our results, we performed an additional experiment with an HLA-A02-restricted HIV-specific CTL targeting a different Gag epitope (FLGKIWPSHK). We again observed significant impairment of infected-cell elimination by CTL that had been pre-treated with romidepsin and SAHA (**Fig. 6F**). The effect of panobinostat was not significant in this assay, though a lesser proportion of infected targets were killed at each of the clone∶target ratios. Thus, even transient exposures to pharmacologically relevant doses of HDACis impair the abilities of CD8^+^ T cells to eliminate HIV-infected target cells. As with other measures of T-cell function reported above, the impairment of CTL elimination of infected cells was particularly severe following treatment with romidepsin at the doses tested.

**Figure 7:**
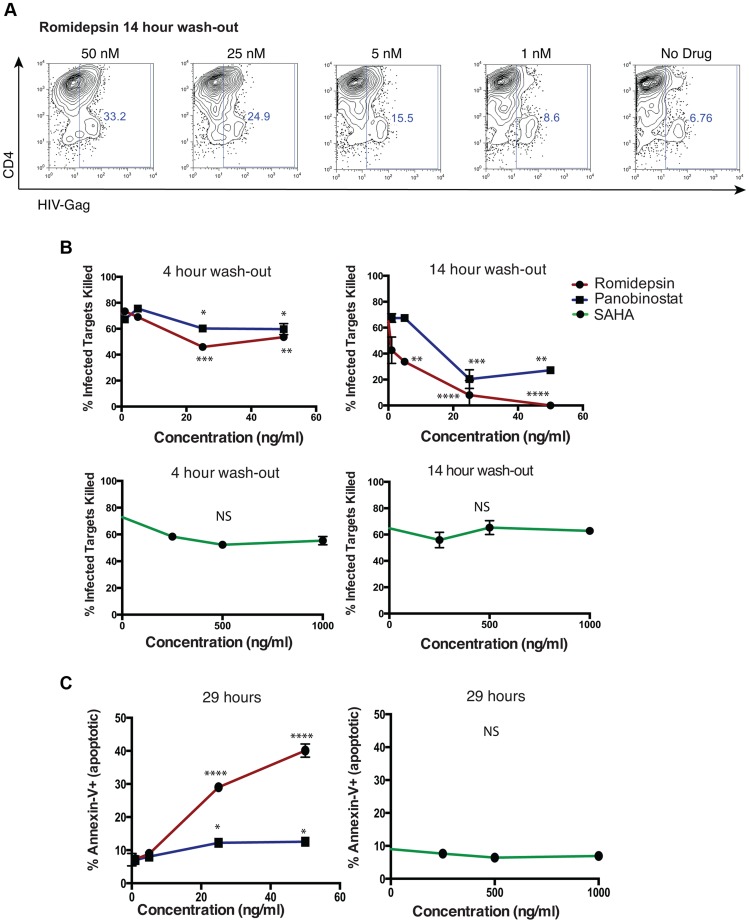
Impairment of CTL killing is sustained for at least 14 hours after removal of romidepsin or panobinostat. An HIV-Gag-specific CTL clone was treated with panobinostat, romidepsin, or SAHA at the indicated doses for 5 hours, or maintained as an untreated control. Cells were then washed two times, resuspended in R10–50, and cultured for 4 or 14 hours. These CTL were then co-cultured with autologous HIV-infected CD4^+^ T-cells at a clone∶target ratio of 1∶10 for 8 hours, and levels of infection were measured as described above (see Fig. 4 legend). **A**. Shown are flow cytometry plots gated on the viable (FSC/SSC) CD8^−^ population, and depicting HIV-Gag staining (x-axis) by CD4 staining (y-axis). For the plots show, CTL that had been treated with romidepsin were given a 14 hour wash-out period prior to co-culture with infected cells. **B**. Shown are summary flow cytometry for 4 hour wash-outs (left panels) and 14 hour wash-outs (right panels). Each condition was tested in duplicate. Error bars represent SEM. **C**. Portions of the CTL used in the above elimination assays were maintained in culture. At the conclusions of the 14 hour wash-out elimination assay, 29 hours in total since addition of drugs, the frequencies of dead (7-AAD^+^) and apoptotic (Annexin-V^+^) CTL were measured by flow cytometry. Shown are % Annexin-V^+^ following treatment with the indicated doses of drugs. Error bars represent SEM. P values were calculated by two-way ANOVA with Dunnett's multiple comparison test (comparing to the no drug control) * p<0.05, ** p<0.01, *** p<0.001, **** p<0.0001.

In an *in vivo* setting, one could envision a scenario where exposure to HDACis induces expression of latent HIV, but then clearance of the drug occurs, potentially allowing CTL to recover functionality and eliminate these unmasked target cells. To model this, we performed an additional series of viral elimination assays where we pre-treated CTL with HDACis for 5 hours, then washed to remove these drugs, and cultured CTL in fresh medium for 4 or 14 hours prior to a 10 hour co-culture with infected target cells (**Fig. 7A**). At the high clone∶target ratio of 1∶10 used in this assay we observed relatively modest impairment of viral elimination in the 4 hour wash-out experiment by romidepsin and panobinostat (mean ± SD % infected targets killed, 73.0±1.3% - no treatment versus 53.5±3.0% – 50 ng/ml romidepsin, p = 0.0013; 60.2±6.1% - 50 ng/ml panobinostat, p = 0.016). We did not observe significant impairment of viral elimination by SAHA in this experiment; 55.4±3.1 – 1,000 ng/ml SAHA, p = 0.07) (**Fig. 7B**). In the 14 hour wash-out experiment we observed greater impairment of target cell killing in the romidepsin and panobinostat treated samples, as compared to the 4 hour wash-out (mean ± SD % infected targets killed, 64.8±0.4% no treatment versus 0±0% - 50 ng/ml romidepsin, 27.2±2.3% 50 ng/ml panobinostat). In contrast, we observed a lack of inhibition in SAHA-treated 14 hour wash-out samples (62.8±0.3% 1,000 ng/ml SAHA). The differences in elimination of infected cells between the 4 hour and 14 hour wash-out experiments were significant across the dose ranges tested, by two-way ANOVA, for romidepsin (p = 0.0187) and for panobinostat (p = 0.0231). These differences for SAHA were not significant (p = 0.065), and, in contrast to romidepsin and panobinostat, the trend for SAHA was towards improved elimination of infected cells following the rest period.

Based on our data from **Figs. 1** **and** **2**, we postulated that the exacerbation, rather than recovery, of CTL impairment over the 14 hour wash-out period was due to the progressive loss of viability of cells following romidepsin or panobinostat treatment, even following HDACi removal. In order to test this, we had maintained CTL in culture in parallel to these elimination assays. Contemporaneously with the termination of elimination assays, we assessed the viability of CTL in these cultures by assessing the frequencies of apoptotic (Annexin-V^+^) and dead (7-AAD^+^) cells by flow cytometry (**Fig. 7C**). We observed the dose dependent induction of apoptosis (Annexin-V^+^) in CTL that had been exposed to romidepsin and, to a lesser extent, panobinostat. Thus, in-line with our previous results, rather than allowing for recovery of CTL function, a wash-out period exacerbated the impairment of infected-cell elimination by romidepsin and panobinostat. In contrast, exposure to SAHA at these doses was not associated with the induction of apoptosis.

### Visualizing the Effects of HDAC Inhibitors on CTL Killing in 3D Collagen Matrices by Time-Lapse Microscopy

**Figure 8:**
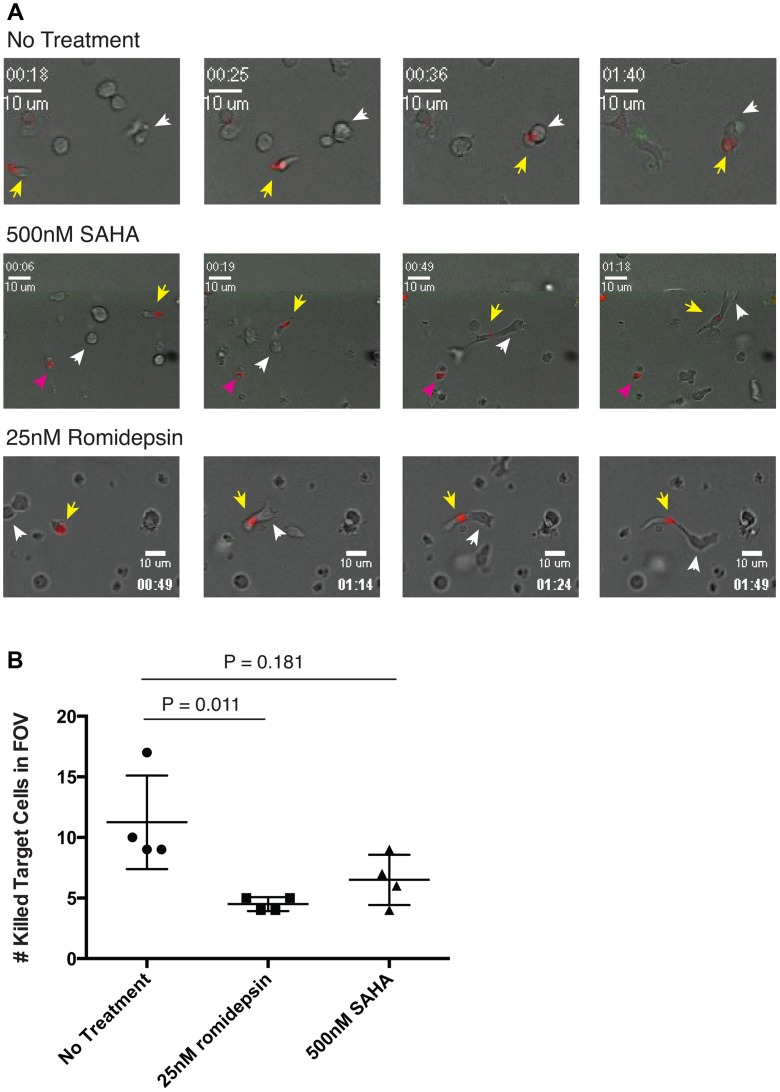
Time-lapse microscopy of CTL killing of peptide pulsed BLCL target cells in 3D collagen matrices. An HIV-Gag-SLYNTVATL-specific CTL clone was labeled with Alexa-Fluor555 conjugated cholera toxin subunit B either cultured with 500 nM SAHA or 25 nM romidepsin for 20 hours, or maintained as an untreated control. These effector cells were combined with SLYNTVATL peptide pulsed target cells, matched on the restricting allele, in a collagen matrix medium containing sytox green viability dye. These mixtures were then plated in three separate wells of an 8-well cover slip and imaged by time-lapse brightfield and fluorescent microscopy. **A**. Shown are representative fields of view from the no treatment (upper panel), 500 nM SAHA (middle panel), and 25 nM romidepsin (lower panel) conditions advancing in time from left to right. Time stamps are given in hh∶mm format. Clones described in the results are indicated with yellow arrows and killed target cells are indicated with white arrows in the upper right panel. **B**. The number of killed (sytox green positive) BLCL at T = 20 minutes were counted in each of the four fields of view acquired for each condition. Each field of view is plotted as a single point on the graph along with means and SD. P values were calculated by the Kruskal-Wallis test. The reported values are corrected for multiple testing using Dunn's multiple comparison test.

To effectively eliminate HIV-infected target cells *in vivo*, CTL need to migrate through the extracellular matrix to catch and kill moving targets. We visualized the effects of pharmacologically relevant doses of romidepsin and SAHA on the killing activity of an HIV-Gag-SLYNTVATL-specific CTL in a 3D collagen matrix designed to approximate this *in vivo* environment as has been previously described [45]. CTL were membrane-labeled with Alexa-Fluor555-conjugated cholera toxin B and pre-treated with the indicated drugs for 24 hours, or maintained as no-treatment controls. These effectors were then mixed with peptide-pulsed BLCL expressing the restricting class I allele in a collagen matrix and visualized by time-lapse brightfield and fluorescence microscopy. CTL are identified in these images by fluorescence from the Alexa-Fluor555 label (red in images), and dead cells acquire fluorescence by permitting a sytox green dye in the medium to pass through their disrupted membranes and bind to genomic DNA (green in images). Untreated CTLs migrated through the collagen matrix and efficiently killed target cells. Images from a representative field of view are shown in the top panels of **Fig. 8A** and the corresponding movie is given in **Supporting Movie S1**. The yellow arrow follows a migrating CTL that engages and kills a target cell marked with a white arrow. In total, three CTL∶target cell engagements and kills are observed in this field of view within 1 hour 40 minutes. Killed target cells appear green due to the ability of sytox dye to pass through their compromised membranes and bind to nuclear DNA. For the CTL treated with romidepsin or SAHA we observed less efficient killing of target cells. The images in the middle panel of **Fig. 8A** correspond to **Supporting Movie S2** and follow a SAHA-treated CTL (yellow arrow) that migrates and engages with a target cell (white arrow). The CTL fails to kill this target and instead is dragged through the collagen until the target cell pulls away and escapes out of frame. In **Supporting Movie S2** one can see that this CTL subsequently engages with a second target cell that it also fails to kill. Two other CTL are observed in the two right panels and, in contrast to the no treatment condition, neither of these is associated with a killed target cell. The lower panel corresponds to **Supporting Movie S3** and shows a similar event observed in the romidepsin condition where a CTL (yellow arrow) engages with a target cell (white arrow) but fails to kill it. The target cell pulls the CTL along and then escapes **Fig. 8A**. In-line with previous experiments, we did also observe an increased frequency of dead/apoptotic CTL (non-motile, small condensed cytoplasm) in association with romidepsin treatment (**Fig. 8B**, middle panel, pink arrow). The total numbers of killed target cells following 20 minutes of imaging were counted in four fields of view (FOV) each for the three treatment conditions. Overall, we observed significantly fewer killing events in the wells containing CTL that had been treated with romidepsin (p = 0.011), but no significant effect of SAHA treatment (p = 0.181) **Fig. 8B**. Thus, treatment with romidepsin impairs the ability of CTL to kill peptide-pulsed target cells in a 3D model of the extracellular environment. The two modes of romidepsin-induced impairment supported by the data presented in previous figures – functional impairment of viable cells, and delayed onset apoptosis – were directly visualized in this experiment with both an apoptotic and a viable but ineffective CTL present in the romidepsin FOV presented in **Fig. 8B**.

## Discussion

In this study we explored the potential for HDACi latency-reversing drugs to impact upon multiple functions of virus-specific CTL. We observed that romidepsin, panobinostat, and SAHA all rapidly suppressed IFN-γ production from virus-specific CD8^+^ T-cells. The proliferation of both CD8^+^ and CD4^+^ T-cells in response to either CMV-pp65 or HIV-Gag peptide pools was abrogated by treatment with romidepsin, while the effects of SAHA and panobinostat on proliferation were only significant for the CD8^+^ T-cell response to CMV-pp65. Critically, each of these HDACis also impaired the ability of HIV-specific CTL to eliminate infected CD4^+^ and peptide pulsed BLCL as measured in liquid culture and collagen matrices respectively.

The overall data support the idea that there are mechanisms both involving and independent of losses in T-cell viability at play, and that these make differential contributions depending on the functional assay being tested, drug concentrations, exposure/rest times, and the type of effector cell (*ex vivo* T-cells, or *in vitro* expanded CTL clone). The data presented in **Figs. 1** and **2** clearly demonstrate that romidepsin and, to a lesser extent, panobinostat are disproportionately toxic to activated T-cells as compared to their resting counterparts. However, data presented in **Fig. 3** demonstrate that all 3 HDACis suppress IFN-γ production from viable T-cells. In the case of romidepsin, our data support the idea that these two modes of suppression are linked, with early suppression of functionality followed by later onset of apoptosis. However, in the case of SAHA, functional suppression appears to occur in the absence of any impact on cell viability. Treatment with panobinostat appears to be intermediate between these two situations, depending also upon the exposure concentration.

We propose that the net effects of HDACi treatment on HIV-specific T-cells in the majority of our functional assays involved contributions from both of these modes of suppression, particularly in the case of romidepsin. For example, the impaired abilities of romidepsin-treated CTL to eliminate HIV-infected target cells over 16 hour co-cultures likely resulted both from rapid suppression of CTL function (CTL are capable of killing target cells within 2–10 minutes [46]–[48]) as well as apoptosis at later time-points post-exposure. This is supported by the observed exacerbation of this impairment following a 14 hour romidepsin wash-out period (**Fig. 7**). This is also directly visualized in **Fig. 8A**, and **Supporting Movie S3**. Here, following romidepsin treatment, apoptotic CTL are visible, but a CTL also remains viable (excludes sytox dye) throughout the 5∶34 (h∶mm) imaging, while failing to kill a target cell that it engages from 1∶03 to 2∶26. These two modes of HDACi-induced CTL impairment – inhibition of function in viable T-cells and reduction in T-cell viability (with the former leading to the latter in some cases) – have the potential to have different impacts in a therapeutic setting. It may be possible to mitigate the impact of transient CTL impairment on flush-and-kill eradication strategies by designing dosing schedules that target a temporal therapeutic window, whereby either HIV antigen expression occurs before CTL are substantially impaired, or persists while CTL are given time to functionally recover. A better understanding of the kinetics and durability of HIV antigen presentation from reactivated latently-infected cells is required to evaluate the plausibility of this approach. On the other hand, losses in viability of activated CTL, as we observed *in vitro* with romidepsin and, to a lesser extent, panobinostat, could result in an irreversible impairment of virus-specific cellular immune responses, particularly if HIV-specific T-cell responses are primed first, for example by therapeutic vaccination. Thus, our data suggest that the potential for a given HDACi to impair CTL function both in the first few hours of treatment when viability is intact, and over longer time-lines by the gradual induction of cell death should be considered in designing flush-and-kill approaches.

Our findings are limited to *ex vivo* and *in vitro* experiments, and the extent to which HDACis impact CTL function in HIV-infected patients is presently unknown. On one hand, the clearance of drugs *in vivo* may serve to mitigate some of the effects of HDACis on HIV-specific CTL, although we attempted to account for this by washing out drug in most assays. On the other hand, our *in vitro* experiments incorporated only single doses of HDACis whereas the SAHA and panobinostat clinical trials have incorporated repeated dosing. This could potentially exacerbate any effects, particularly if these drugs cause lasting changes to the T-cell functional profile, or compromise the survival of T-cells. In the CLEAR trial, for example, patients received panobinostat on days 1, 3, and 5 every other week for 8 weeks. An additional challenge to predicting whether a therapeutic window between latency reversal and CTL inhibition exists is that infected cell elimination assays, which utilized highly functional CTL clones, at relatively high effector∶target ratios in most experiments, and activated target cells expressing high levels of HIV-Gag, can be interpreted as an idealized environment for CTL killing of target cells. Thus, it is reasonable to speculate that even a subtle difference in killing efficiency observed in our *in vitro* assays may manifest as a critical difference *in vivo* where CTL are likely to be exhausted or otherwise functionally impaired, and where CTL encounters with reactivated target cells are rare. Following from this, while we did not observe significant impairment of infected cell killing at 1 nM or 5 nM of panobinostat, we cannot rule out that more subtle impairments in function may lead to reductions in abilities of CTL to eliminate exposed natural reservoir cells *in vivo*. Based on the magnitudes of the effects observed *in vitro*, along with consideration of the pharmacokinetic and pharmacodynamic properties of these drugs, at the dosing regimens being taken forwards into HIV clinical trials we propose that HDACis are differentially likely to impact upon relevant T-cell functions *in vivo*, with the following hierarchy: romidepsin>panobinostat>SAHA. Ultimately, however, we hope that the primary outcome of our study will be to motivate the incorporation of assays measuring *ex vivo* T-cell function into ongoing and planned HDACi clinical trials, and that immunosuppression will be considered as a potential factor limiting the effectiveness of any observed outcomes.

We must also highlight, in a more general sense, the potential risk of treating HIV-infected subjects, whose immune systems do not fully recover even with ART, with HDACis. In addition to the *ex vivo* and *in vitro* data presented in the current study, a number of studies have observed *in vivo* immunosuppressive activities of HDACis, including romidepsin and SAHA [16]–[20]. While opportunistic infections have not been reported in HIV-infected subjects receiving SAHA and panobinostat, this is a greater concern for romidepsin. We observed greater impairment of CTL function, and toxicity with romidepsin treatment as compared to panobinostat and SAHA at the doses tested. Lymphopenia is also known to be a common side-effect of romidepsin treatment, and serious and sometimes fatal infections, including pneumonia and sepsis, have been reported in clinical trials in oncology settings [49]. Notably though, these side-effects were observed at higher doses than those planned for HIV eradication trials. The incorporation of immunological end-points as important safety parameters in the early stages of testing romidepsin in HIV-infected subjects would help to address concerns regarding a potential impact on the immune system in general. The possibility that romidepsin or other HDACis could impair HIV-specific T-cell responses *in vivo* should also be ruled out before any therapy interruptions are contemplated as part of eradication trials, given the importance of CTL in containing any rebound viremia.

The HIV-specific cellular immune response, in addition to playing a vital role in the ongoing health of HIV-infected individuals, stands to make a powerful contribution to HIV eradication strategies. Thus we would make a case that a critical stage in evaluating potential latency reversing drugs to be used in CTL-based flush-and-kill strategies should be to test the effects of these drugs on the functions of HIV-specific T-cells. This information can both be used to help prioritize classes of candidates, and to select optimal drug candidates from within a class. We would also suggest that this should be extended to flush-and-kill strategies focused on other immune effectors. Notably, romidepsin has also been reported to both suppress the cytotoxic activity and to drive apoptosis of NK cells, which are another potential key immune effector in flush and kill strategies [24]. Information regarding how a drug influences CTL and other immune effectors should be generated along with other parameters such as EC_50_ and toxicity in order to prioritize candidates. Through screening efforts, or rational design based on an improved understanding of the mechanisms by which HDACis exert their immunosuppressive effects, it may also be possible to identify HDACis that retain the ability to induce the expression of latent HIV without impairing the ability of CTL or other immune effectors to eradicate these unmasked targets.

## Materials and Methods

### Ethics Statement

HIV-infected individuals were recruited from the Maple Leaf Medical Clinic in Toronto, Canada through a protocol approved by the University of Toronto Institutional Review Board and from the Boston area (United States) under a protocol approved by the Institution Review Board at the Massachusetts General Hospital. Secondary use of the samples from Toronto was approved through the Massachusetts General Hospital Institutional Review Board. All subjects were adults, and gave written informed consent. Clinical data for study subjects are given in Table 1.

**Table 1:** Clinical characteristics of study subjects.

ID	Viral Load	CD4 count	ART Regimen	Treatment Duration
653116	20	624	Emtricitabine, Tenofovir, efavirenz	81 months
514023	20	1258	Emtricitabine, Tenofovir, atazanavir, ritonavir	92 months
128450	40	635	Emtricitabine, Tenofovir, Raltegravir	23 months
198605	50	1234	Combivir, Tenofavir	66 months
296260	20	671	Emtricitabine, Tenofovir, efavirenz	62 months
379080	48	643	Atripla	53 months
394747	48	711	Atripla	39 months
403998	20	738	Atripla	48 months
409231	50	795	Tenofavir, Kaletra, Abacavir	19 months
498553	48	1128	Ritonavir, Atazanavir, Truvada	20 months
557014	20	1426	Truvada, Raltegravir	35 months

### Latency Reversing Drugs

Romidepsin was purchased from Selleckchem. An 18.5 mM stock was prepared in 100% DMSO (hybrimax, Sigma). This was diluted 3,700× in PBS to give a working stock of 5 µM in 0.03% DMSO. SAHA was purchased from Sigma and dissolved to 10 mM in 100% DMSO. This was diluted to a working stock of 1 mM in PBS (10% DMSO in working stock). Panobinostat was obtained from Selleckchem as a 10 mM stock in DMSO. This was diluted 500× in in PBS to give a working stock of 20 µM in 0.2% DMSO. ALT-803 is an IL-15 superagonist (IL-15SA) produced by Altor Bioscience Corporation [50], [51]. IL-15 has previously been shown to increase the magnitude of HIV-specific T-cell responses as detected by IFN-γ ELISPOTS through a mechanism involving STAT1, STAT3, STAT4, and STAT5 [52], [53], and thus allowed for a control for cell responsiveness to soluble factors that impact cellular functions. ALT-803 was obtained from Altor Bioscience Corporation at 1.38 mg/ml in PBS, and was used at a working stock of 50 µg/ml. The concentrations of HDACis used in experiments were selected to be pharmacologically relevant to current clinical trials. The mean clinical C_max_ of panobinostat at the 20 mg p.o. dosing regimen of the ongoing CLEAR trial is 40 nM [54], the steady-state plasma concentration is 15 to 22 nM (Novartis Investigator's brochure), and the EC_50_ values for *in vitro* HIV activation in cell line models of latency has been reported to range from 10–16 nM [11], [55]. The mean C_max_ of SAHA given at 400 mg p.o. as in the two latency reactivation trials that have been conducted is 1 µM [56], and the reported EC_50_ values for *in vitro* HIV activation in cell line and primary cell models of latency range from 885–3,950 nM [11], [55], [57]. The mean clinical C_max_ values for of romidepsin in the intermediate and high dosing arms of the upcoming dose escalation study in HIV-infected subjects (ClinicalTrials.gov Identifier: NCT01933594) are: 2 mg/m^2^, C_max_ 69.5±32.6 nM, and (5 mg/m^2^, 178.1±32.6 nM), and the reported EC_50_ values for *in vitro* HIV activation in a cell line and primary cell models of HIV range from 3–4.5 nM [55], [57].

### T-Cell Viability Assay

PBMC were obtained from HIV-negative buffy coats, or from cryopreserved leukophersis samples from HIV-infected subjects (as indicated). CTL clones were taken from long-term *in vitro* cultures (restimulated bi-weekly). Cells were plated at 200,000 cells/well (PBMC) or 50,000 cells/well (CTL clones) in 200 ul of RPMI-1640+10% FBS (R10) medium (PBMC) or R10+50 U/ml IL-2 (CTL clones) in 96-well round-bottom plates. Where indicated, cells were stimulated with 1 µg/ml each of anti-CD3 (OKT3 clone) and anti-CD28 (CD28.2 clone), both from eBioscience, for 48 hours prior to HDACi addition. HDACis were added at the indicated concentrations with all conditions tested in triplicate. Cells were cultured at 37°C, 5% CO_2_ for 4 the indicated periods of time. Where indicated, HDACis were washed out with 2×250 µl medium and cells were replated in fresh medium. At the time of harvest, cells were stained with Annexin-V-Fitc, 7-AAD, anti-CD4 pacific blue (BioLegend), and anti-CD8 alexa-fluor 700 (BioLegend) using the FITC Annexin V Apoptosis Detection Kit with 7-AAD (BioLegend), washed, fixed in 4% paraformaldehyde and then analyzed immediately on an LSR-II flow cytometer.

### PMA/Ionomycin Intracellular Cytokine Staining Assays

PBMC were plated at 100,000 cells/well in 96-well round bottom plates in R10 medium and HDACis were added at the indicated concentrations. Following 4 hours of exposure, cells were washed with 2×250 µl medium and then cultured with 200 µl fresh R10 medium+1/500 dilution of the 500× PMA/ionomycin cell stimulation cocktail (eBioscience) in the presence of 1 µg/ml Brefeldin A (BD). Following a 5 hour stimulation period, cells were split and stained with either: i) anti-CD4 pacific blue (BioLegend), and anti-CD8 alexa-fluor 700 (BioLegend), permeabilized (cytofix/cytoperm, cytoperm/wash, BD) and stained intracellularly with anti-IFN-γ FITC (to measure IFN-γ production) ii) Annexin-V-Fitc, 7-AAD, anti-CD4 pacific blue (BioLegend), and anti-CD8 alexa-fluor 700 (BioLegend) using the FITC Annexin V Apoptosis Detection Kit with 7-AAD (BioLegend) (to measure viability). Cells were fixed % paraformaldehyde and then analyzed immediately on an LSR-II flow cytometer.

### Generation of T-Cell Clones

PBMCs were stimulated with optimal CD8 T-cell epitopes for 6-hours, enriched for antigen-specific cells using the IFN-γ secretion Detection and Enrichment Kit (Miltenyi), and cloned at limiting dilution on irradiated feeder cells as has been previously described [58]. Clones were selected from 96-well plates at dilutions where no more than 1 in 5 wells displayed growth and screened for specificity by IFN-γ ELISPOT. Specific clones were expanded on irradiated feeder cells.

### IFN-γ ELISPOT

ELISPOT assays were performed as previously described [59]. CD8^+^ T-cell clones were plated at 10,000 cells/well with 50,000 autologous EBV-transformed B cell lymphoblastoid cell lines (BLCL) as target cells. Peripheral blood mononuclear cells (PBMC) were plated at 100,000 cells/well. Peptides representing optimal HIV CD8^+^ T-cell epitopes were used at final concentrations of 1 µg/ml/peptide, while the pool of overlapping CMV-pp65 15mer peptides (JPT peptide technologies) was used at 1 µg/ml/peptide. Cells were incubated for 16 hours with peptide in ELISPOT plates before beginning development. The number of specific spot-forming cells (SFC) was calculated by subtracting the background number of spots in the negative control (DMSO only) control wells from the number of spots in the experimental well. For assays performed on CTL clones values are reported as SFC per thousand CTL clone cells. For assays performed on PBMC values are reported as SFC per million PBMC.

### Proliferation Assays

PBMC from 12 ARV-treated HIV-infected subjects were thawed and labeled with 0.5 µM CFSE in PBS. Cells were plated at 100,000 cells/well in 96-well round bottom plates in R10. Triplicate wells were prepared for each experimental antigen/drug combination, and outside wells of the plate were filled with PBS. HDACis or ALT-803 were added in 50 µl of R10 to final concentrations of: ALT-803 72 ng/ml, romidepsin 25 nM, panobinostat 25 nM, SAHA 500 nM. These concentrations were selected to fall below the Cmax concentrations in ongoing and planned clinical trials (see results). No treatment controls were prepared in parallel with 50 µl of R10. Cells were incubated at 37°C, 5% CO_2_ for 4 hours. Pools of overlapping 15mer peptides spanning CMV-pp65 (Positive Control Pool Pepmix HCMV(pp65), JPT Peptide Technologies) and 18mer peptides overlapping by 10 amino acids spanning HIV-Gag were obtained from the MGH peptide core facility and added to final concentrations of 1 µg/ml/peptide. After 48 hours of further incubation, cells were washed 2× with 200 µl of R10 and resuspended in 200 µl of R10 supplemented with 20 U/ml IL-2 (NIH AIDS reagent program). Where indicated, the Gag peptide pool was re-added along with this fresh medium at a concentration of 1 µg/ml/peptide. Following an additional 5 days of culture (7 days total) cells were stained with anti-CD4 pacific blue, anti-CD8 alexafluor700, and anti-CD3 PE-CY7 (all antibodies from BioLegend) and analyzed by flow cytometry on an LSRII instrument. Data were analyzed using Flowjo software (Treestar).

### Production of HIV

The HIV molecular clone JR-CSF was obtained from the NIH AIDS Reagent Program (www.aidsreagent.org). Viral stocks were produced by transfection of 293T cells using Fugene HD (Promega) following the manufacturer's instructions. Supernatants were harvested 48 hours post-transfection, centrifuged at 1,000×g for 5 minutes to pellet cell debris, and filtered through a 400 µM membrane (Steriflip, Millipore). Viral titers were determined by titration on primary cells by the Ragon Institute Virology Core.

### ACH2 Latency Reversal Assays

The chronically HIV-infected ACH2 cell line was maintained in R10 medium. Cells were plated at 100,000 cells/well in 96-well round-bottom plates. Latency reversal drugs were added at the indicated concentrations and incubated for 4 hours. Cells were then washed two times to remove drugs, replated in fresh R10 medium, and cultured for an additional 20 hours. Cells were fixed and permeabilized using cytofix/cytoperm and perm/wash reagents (BD) and then stained intracellularly with anti-HIV-Gag KC57-RD1 (Beckman Coulter) diluted 1/100 in perm/wash buffer. After washing to remove unbound antibodies, cells were analyzed on a FACSCalibur instrument (BD).

### HIV Elimination Assays

HIV-Gag SLYNTVATL and FLGKIWPSHK specific CD8^+^ T-cell clones were obtained from two different HIV-infected elite controller subjects and maintained as previously described [58], [60]. The specificities and cytotoxic potential of these clones were confirmed two days prior to performing each elimination assay experiment by measuring degranulation in response to cognate peptide stimulation by a CD107a-staining flow cytometry method [61]. On this same day, HLA-A02^+^ PBMC from HIV-uninfected subjects were thawed and enriched for CD4^+^ T-cells by negative selection (Easysep, Stemcell Technologies). These CD4^+^ T-cells were stimulated with 1 µg/ml each anti-CD3 OKT3 and anti-CD28.2 antibodies (eBioscience) in R10 supplemented with 50 U/ml IL-2 (NIH AIDS Reagent Program) for 48 hours. These activated target cells were infected with HIV JR-CSF using a previously described magnetofection method [62]. Infection levels were monitored by flow cytometry staining for CD4 (BioLegend) and intracellularly for HIV-Gag (KC57 clone, Beckman Coulter). Targets were determined to be ready for use in the elimination assay when they were 20% Gag^+^ by flow cytometry. At this time-point, CTL clones were split into equal aliquots and cultured in R10+50 U/ml IL-2 supplemented with HDACis and ALT-803 as indicated. Following 4 hours of incubation in 5% CO_2_ at 37°C, CTL were washed with 3× with 1 ml R10. Target CD4^+^ T-cells were plated at 50,000 cells/well in 96-well round-bottom plates, and CTL were added at the indicated effector∶target ratios. Each condition was set up in triplicate. Co-culture was allowed to proceed for 16 hours at 37°C, 5% CO_2_. Cells were then surface stained with anti-CD8-Fitc and anti-CD4-APC (BioLegend), permeabilized (cytofix/cytoperm, BD) and stained intracellularly with anti-HIV-Gag-PE at 1/100 dilution (KC57 clone, Beckman Coulter). Cells were then fixed with 4% formalin in PBS and analyzed on an LSR-II flow cytometer instrument (BD Biosciences). Data were analyzed using Flowjo software (Treestar).

### Time-Lapse Microscopy of CTL Killing in 3D Collagen Matrices

Following confirmation of functional activity (see above), an HIV-Gag-specific CTL clone was labeled with 5 µg/ml cholera toxin subunit B AlexaFluor 555 (Life Technologies) in 0.5 ml R10+50 U/ml IL-2 for 10 minutes at 37°C. Cells were washed, split into equal aliquots in R10+50 U/ml IL-2 and treated with HDACi drugs at the indicated concentrations for 14 or 24 hours. HLA-A02^+^ BLCL were then pulsed with 100 ng/ml SLYNTVATL Gag peptide for 30 minutes, and washed. CTL and BLCL were each resuspended to final concentrations of 2.7×10^6^ cells/ml in R10+50 U/ml IL-2. 3D collagen time-lapse microscopy was performed as previously described [45].

### Statistical Analysis

Statistical analyses were performed using Prism software (Graphpad). The statistical tests used to calculate p values are indicated in the corresponding figure legends.

## Supporting Information

Figure S1 **Effects of HDACis on the viability of PBMC CD8^+^ and CD4^+^ T-cells from HIV-infected subjects, and on HIV-specific CTL clones.** **A**. Cryopreserved PBMC from the ARV-treated HIV-infected subjects OM292 and OM265 were thawed and then treated with HDACi drugs at the indicated concentrations for 6 hours. Cells were harvested at the indicated time-points 21 hours and stained with 7-AAD (stains DNA of dead cells), CD4, CD3, and CD8. Conditions were tested in triplicate. Shown are summary data depicting means ± SEM. P values were calculated by two-way ANOVA with Dunnett's multiple comparison test (comparing to the no drug control) * p<0.05, ** p<0.01, *** p<0.001, **** p<0.0001.
(EPS)

Movie S1 **Time-lapse microscopy of untreated CTL with peptide pulsed BLCL target cells.** Shown is a time-lapse microscopy movie corresponding to the images depicted in the upper panel of **Fig. 7A**.
(MOV)

Movie S2 **Time-lapse microscopy of SAHA-treated with peptide pulsed BLCL target cells.** Shown is a time-lapse microscopy movie corresponding to the images depicted in the middle panel of **Fig. 7A**.
(MOV)

Movie S3 **Time-lapse microscopy of romidepsin-treated with peptide pulsed BLCL target cells.** Shown is a time-lapse microscopy movie corresponding to the images depicted in the lower panel of **Fig. 7A**.
(MOV)
