# Correction: Histone Deacetylase Inhibitors Impair the Elimination of HIV-Infected Cells by Cytotoxic T-Lymphocytes

**DOI:** 10.1371/journal.ppat.1004572

**Published:** 2014-11-21

**Authors:** The PLOS Pathogens Staff

## Notice of Republication

This article was republished on November 7, 2014, to correct errors in the Figure Legends of the XML version of the article. These errors were introduced during the typesetting process and have been corrected.
